# Metabolic transition from childhood to adulthood based on two decades of biochemical time series in three longitudinal cohorts

**DOI:** 10.1093/ije/dyaf026

**Published:** 2025-03-26

**Authors:** Ville-Petteri Mäkinen, Mika Kähönen, Terho Lehtimäki, Nina Hutri, Tapani Rönnemaa, Jorma Viikari, Katja Pahkala, Suvi Rovio, Harri Niinikoski, Juha Mykkänen, Olli Raitakari, Mika Ala-Korpela

**Affiliations:** Faculty of Medicine, Systems Epidemiology, Research Unit of Population Health, University of Oulu, Oulu, FI-90014, Finland; Biocenter Oulu, University of Oulu, Oulu, FI-90014, Finland; Department of Clinical Physiology, Tampere University Hospital, Tampere, FI-33521, Finland; Finnish Cardiovascular Research Center Tampere, Faculty of Medicine and Health Technology, Tampere University, Tampere, FI-33014, Finland; Finnish Cardiovascular Research Center Tampere, Faculty of Medicine and Health Technology, Tampere University, Tampere, FI-33014, Finland; Department of Clinical Chemistry, Tampere University Hospital, Tampere, FI-33521, Finland; Fimlab Laboratories, Pirkanmaa Hospital District, Tampere, FI-33520, Finland; Faculty of Medicine and Health Technology, Tampere Centre for Skills Training and Simulation, Tampere University, Tampere, FI-33014, Finland; Department of Medicine, University of Turku, Turku, FI-20014, Finland; Division of Medicine, Turku University Hospital, Turku, FI-20520, Finland; Department of Medicine, University of Turku, Turku, FI-20014, Finland; Division of Medicine, Turku University Hospital, Turku, FI-20520, Finland; Research Centre of Applied and Preventive Cardiovascular Medicine, University of Turku, Turku, FI-20014, Finland; Centre for Population Health Research, University of Turku and Turku University Hospital, Turku, FI-20520, Finland; Paavo Nurmi Centre, Unit of Health and Physical Activity, University of Turku, Turku, FI-20014, Finland; Research Centre of Applied and Preventive Cardiovascular Medicine, University of Turku, Turku, FI-20014, Finland; Centre for Population Health Research, University of Turku and Turku University Hospital, Turku, FI-20520, Finland; Research Centre of Applied and Preventive Cardiovascular Medicine, University of Turku, Turku, FI-20014, Finland; Centre for Population Health Research, University of Turku and Turku University Hospital, Turku, FI-20520, Finland; Department of Pediatrics and Adolescent Medicine, Turku University Hospital and University of Turku, Turku, FI-20520, Finland; Research Centre of Applied and Preventive Cardiovascular Medicine, University of Turku, Turku, FI-20014, Finland; Centre for Population Health Research, University of Turku and Turku University Hospital, Turku, FI-20520, Finland; Research Centre of Applied and Preventive Cardiovascular Medicine, University of Turku, Turku, FI-20014, Finland; Centre for Population Health Research, University of Turku and Turku University Hospital, Turku, FI-20520, Finland; Department of Clinical Physiology and Nuclear Medicine, Turku University Hospital, Turku, FI-20520, Finland; Faculty of Medicine, Systems Epidemiology, Research Unit of Population Health, University of Oulu, Oulu, FI-90014, Finland; Biocenter Oulu, University of Oulu, Oulu, FI-90014, Finland; NMR Metabolomics Laboratory, School of Pharmacy, Faculty of Health Sciences, University of Eastern Finland, Kuopio, FI-70211, Finland

**Keywords:** children, puberty, metabolism, longitudinal, cardiovascular risk factor, lipids, amino acids, insulin, inflammation, apolipoprotein B

## Abstract

**Background:**

This is the first large-scale longitudinal study of children that describes the temporal trajectories of an extensive collection of metabolic measures that are relevant for lifelong cardiometabolic risk. We also provide a comprehensive picture on how metabolism develops into mature adult sex-specific phenotypes.

**Methods:**

Children born in 1962–92 were recruited by three European studies (*n* = 20 377 eligible). Biochemical data for ages 0–26 years were available for *n* = 14 958 participants (*n* = 8385 with metabolomics). Age associations for 168 metabolic measures (6 physiological traits, 6 clinical biomarkers, and 156 serum metabolomics measures) were determined by using curvilinear regression. Puberty effects were calculated by using logistic regression of biological sex for pre- and post-pubertal age strata.

**Results:**

Age-specific concentrations were reported for all measures. Nonlinear age associations were typical, including insulin (*R*^2^ = 20.7% ±0.6% variance explained ±SE), glycerol (13.3% ±1.3%), glycoprotein acetyls (40.3% ±1.5%), and branched-chain amino acids (19.5% ±1.6%). Apolipoprotein B was not associated with age (0.7% ±0.4%). Multivariate modeling indicated that boys diverged from girls metabolically during ages 13–17 years. Puberty effects were observed for large high-density lipoprotein cholesterol (*P *=* *8.5 × 10^−288^), leucine (*P *<* *2.3 × 10^−308^), glutamine (*P *<* *2.3 × 10^−308^), albumin (*P *=* *1.7 × 10^−161^), docosahexaenoic acid (*P *=* *5.2 × 10^−50^), and sphingomyelin (*P *=* *4.4 × 10^−90^).

**Conclusion:**

Novel associations between emerging cardiometabolic risk factors, such as amino acids and glycoprotein acetyls, and growth and puberty were observed. Conversely, apolipoprotein B was stable, which favors its utility for early assessments of lifetime cardiovascular risk.

Key Messages

- Maturation of childhood metabolism into adult sex-specific metabolic profiles was investigated by using biochemical time-series data of the same individuals from infancy to young adulthood.
- Novel information on the temporal trajectories of lipoprotein subclasses, glucose metabolism intermediates, branch-chain amino acids, and glycoprotein acetyls was published along with new population-based longitudinal descriptions of clinically important cardiometabolic biomarkers such as insulin, low-density lipoprotein cholesterol, and apolipoprotein B.
- This study highlights the importance of age- and sex-specific considerations for predicting future population disease trends from childhood metabolomics data and it provides a new resource for assessing the metabolic health of individual children at different developmental stages.

## Introduction

Cardiometabolic risk factors (insulin, glucose, and lipoprotein lipids) vary in human populations [1–8] and metabolic stratification in childhood is associated with adverse health consequences in adulthood [9–11]. It is thus important to describe the temporal trajectories of metabolic measures from birth through to adulthood so that successive generations can be assessed for health risks and early societal interventions developed where necessary. In particular, accurate analyses on the dominant effect of age are necessary to achieve better health outcomes [12].

New high-throughput technologies that quantify a large number of biochemical traits have emerged recently, notably nuclear magnetic resonance (NMR) metabolomics, which have had a profound impact on large-scale epidemiological studies of cardiometabolic risk factors, including those in children [7, 8, 13–17]. However, robust information about the temporal early-life patterns of many new measures remains limited compared with the established clinical biomarkers. For example, branched-chain amino acids and glycoprotein acetyls are predictive of adverse cardiometabolic outcomes [18–22] and previous studies have revealed associations with childhood obesity [13] but there is less information about the temporal trajectories associated with growth and maturation. The goal of this study is to provide a comprehensive picture on how metabolism develops in children as seen through circulating biomarkers and how the concentrations diverge between boys and girls during puberty.

We analyse biochemical data from 14 958 participants across three European cohorts. We start from established biomarkers such as insulin and clinical lipids, as they are proven indicators of overall metabolic health status and relevant for the obesity pandemic and high cardiovascular risk. Next, we provide novel information on metabolic measures, obtained by using NMR metabolomics, that have been identified as cardiometabolic risk factors in epidemiological studies [14, 18, 20, 23]. Lastly, we use regression modeling to summarize which metabolic measures are the most distinctive of adult vs. prepubertal sex dimorphism and therefore require age-appropriate reference ranges for children if incorporated into public health workflows.

## Methods

The Special Turku Coronary Risk Factor Intervention Project (STRIP) was a randomized–controlled trial that was started in 1990 to investigate whether a favorable diet improved cardiometabolic risk factors [24, 25]. Initial recruitment was 1880 infants aged 5 months (data available from 1105), of whom 1062 went through randomization and 532 remained at age 14 years. In this study, biochemical data collected up to age 26 years are included (standard biochemistry: *n* = 457–923 per visit, 20 visits; metabolomics: *n* = 444–584 per visit, 7 visits).

The Cardiovascular Risk in Young Finns Study (YFS) recruited 3596 children across six peer groups in 1980 in five cities in Finland [26]. In this study, biochemical data measured up to 24 years of age were used. To mitigate batch effects, data were calibrated by using a previously published algorithm [27]. In total, *n* = 3583 participants spread across nine age points were included, with *n* = 284 metabolomics measurements at age 24 years.

The Avon Longitudinal Study of Parents and Children (ALSPAC) is a birth cohort from the South West of England [28–31]. The initial number enrolled in 1991 and 1992 was 13 988, with 913 added to the final sample size of 14 901 (alive after the first year). This study utilizes biochemical data that were collected from before October 2021 (biochemistry *n* = 489–5253 per visit, 8 visits; metabolomics: *n* = 2682–5247 per visit, 4 visits). The study website contains details of data that are available through a searchable data dictionary (URL: http://www.bristol.ac.uk/alspac/researchers/our-data/).

A proportion of participants were lost to follow-up or had incomplete data. The exact numbers for a given variable and time point are provided in Supplementary Table S1 and the overall dataset structure is illustrated in Supplementary Fig. S1 (see online supplementary material for color versions of the figure and table). Height, weight, waist, and blood pressure were obtained by using standard techniques and total triglycerides, total cholesterol, high-density lipoprotein (HDL) cholesterol, insulin, glucose, and C-reactive protein were quantified from blood samples according to standard clinical laboratory techniques [24, 26, 28]. One hundred and fifty-six additional biochemical traits were quantified from blood by using a high-throughput NMR metabolomics platform [15].

We have previously addressed biochemical batch effects in the YFS [27] and in UK Biobank [32]. Here, a batch refers to the samples that were collected from a specific scheduled survey of the cohort. Batch effects create artificial differences between consecutive visits. They arise from variation in sample storage time, handling protocols, and changes in measurement assays. We observed substantial batch effects in the ALSPAC (Supplementary Fig. S2; see online supplementary material for a color version of this figure). Hence, we used the ALSPAC only for investigating sex differences to cancel out the confounding effect.

## Statistical analyses

We used the nroPreprocess() function in Numero R package [33] with default settings to log-transform skewed variables and to standardize data to zero mean and unit variance. Robust means for data subsets were calculated from the standardized values and then reverted back to the original scale and location by using the nroPostprocess() function. Descriptive statistics and regression coefficients were calculated within a bootstrapping framework in which random subsets of the data were repeatedly drawn with replacement and the statistical analysis was repeated for each subset. After bootstrapping, the preprocessing pipeline was applied in reverse to restore the samples to their original value spaces. Confidence intervals and *P*-values were calculated from the restored bootstrap samples. The threshold *P *<* *0.0001 was chosen to highlight the most robust findings.

Curvilinear regression was used for calculating how much of metabolic trait variance was explained by age (Supplementary Fig. S3; see online supplementary material for a color version of this figure). Model fit was confirmed by using visual inspection of the residuals. In some situations, the youngest (or the oldest) age stratum had an outsized influence on the model if the stratum deviated from the rest. The data were insufficient to ascertain how much of the influence was due to biological or batch effects. To solve the problem, we incorporated a cross-validation scheme that added variation from random batch effects into the regression model (Supplementary Fig. S3 caption; see online supplementary material for a color version of this figure). Confidence intervals and *P*-values were calculated within the bootstrapping framework described above.

We applied projections to latent structures to quantify sex dimorphism according to the biochemical and metabolomics data at different ages (Supplementary Fig. S4; see online supplementary material for a color version of this figure). This approach allowed us to leverage the ALSPAC data without confounding from batch effects. The age-specific models were adjusted for differences in the ratio of males and females. Confidence intervals were calculated under the aforementioned bootstrapping framework.

To identify which metabolic variables exhibited sex divergence due to puberty, we used univariate regression modeling for each metabolic measure separately, and before and after puberty. Two sets of logistic regression models of male sex were created for those participants who were ≤13 years old and for those ≥17 years old. We denote the regression coefficient for a metabolic variable as α for the younger and β for the older group. Usually, α is close to zero, as young boys and girls are similar, but the magnitude of β is typically larger due to prominent sex differences in adults (negative if women have a higher measurement than men or positive when vice versa).

## Results

**Figure 1.**
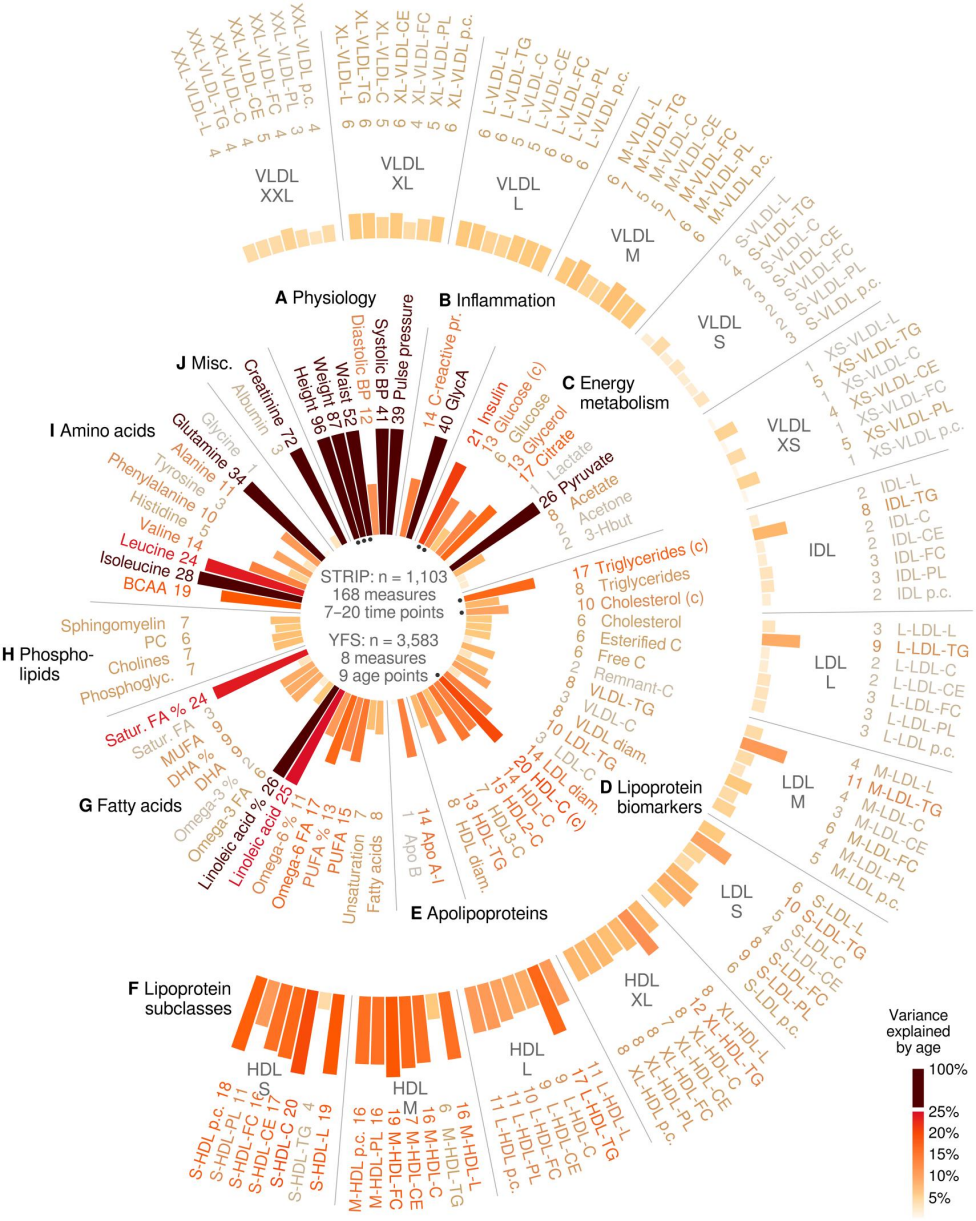
Summary of age associations for 168 metabolic measures. The numbers on the circle indicate percent variance explained by age. The subplot labels refer to the inner circle, except Plot F, which covers the entire outer arc of lipoprotein subclass measures. For most measures, data from the STRIP cohort covered seven time points between the ages of 9 and 26 years. Height, weight, waist, and standard clinical chemistry measures (c) of insulin, glucose, triglycerides, cholesterol, and HDL cholesterol were also available from the YFS cohort and from early childhood onward. Apo, apolipoprotein; BCAA, branched-chain amino acids; BP, blood pressure; DHA, docosahexaeonic acid; FA, fatty acid; GlycA, glycoprotein acetyls; HDL, high-density lipoprotein; IDL, intermediate-density lipoprotein; LDL, low-density lipoprotein; PUFA, polyunsaturated fatty acids; STRIP, Special Turku Coronary Risk Factor Intervention Project; VLDL, very-low-density lipoprotein; YFS, Cardiovascular Risk in Young Finns Study; lipoprotein subclass lipids: L, total lipid; TG, triglycerides; C, cholesterol; CE, cholesterol esters; PL, phospholipids; pc, particle concentration; lipoprotein subclass sizes: L, large; M, medium; S, small; XS, extra small; XL, extra large; XXL, extremely large.

Table 1 is an overview of the results and the age effects are listed in Fig. 1 and Supplementary Fig. S5 (see online supplementary material for a color version of this figure). In the text, we highlight associations that satisfy *P *<* *0.0001 unless otherwise indicated (exact *P*-values are shown in Supplementary Table S2; see online supplementary material for a color version of this table). The intervention in the STRIP caused differences in specific variables (Supplementary Fig. S6; see online supplementary material for a color version of this figure). The models were adjusted for the treatment arm.

**Table 1.** An overview of the cohort participants. The table contains the total numbers of individuals with at least one biochemical measurement that was included in the statistical analyses. Please note that the numbers vary for specific analyses; see Supplementary Tables S1–S4 for further details (see online supplementary material for color versions of these tables).

Variable	ALSPAC	STRIP	YFS
Males	5178 (50.4%)	565 (51.2%)	1755 (49.0%)
Females	5094 (49.6%)	538 (48.8%)	1828 (51.0%)
Age range (years)	0–25.4	0.7–26	3–24
Mean ±SD number of visits per participant	2.4 ± 1.5	11.5 ± 7.1	2.7 ± 1.0

ALSPAC, Avon Longitudinal Study of Parents and Children; SD, standard deviation; STRIP, Special Turku Coronary Risk Factor Intervention Project; YFS, Cardiovascular Risk in Young Finns Study.

Physical growth and development were evident (Supplementary Fig. S7; see online supplementary material for a color version of this figure). Age explained 96% of the body height variance, between 12% and 41% of the blood pressure measures, and 72% of creatinine variance (Fig. 1A and J). Forty percent of the variance in glycoprotein acetyls was explained by age (Fig. 1B). Energy metabolism was associated with growth and development (Fig. 1C): 21% of insulin was explained by age and notable percentages were found for glycerol (13%), citrate (17%), and pyruvate (26%).

Of the clinical lipids (Fig. 1D), HDL cholesterol exhibited the strongest association with age (20%). The association between apolipoprotein B and age was close to zero (1%, *P *=* *0.0047; Fig. 1E). Triglycerides in very-low-density lipoprotein (VLDL) and low-density lipoprotein (LDL) subclasses (Fig. 1F) changed with age but the effect size was modest (≤11%). Intermediate-density lipoprotein (IDL) and LDL cholesterol associations were closer to zero (typically <5%). The smaller HDL subclasses were more age-dependent than the larger (e.g. 8% explained of extra large HDL cholesterol vs. 20% of small HDL cholesterol). Polyunsaturated lipids, particularly omega-6 (17%) and linoleic acid (25%), were partly explained by age (Fig. 1G).

Branched-chain amino acids were age-dependent, including 28% of isoleucine variance explained by age (Fig. 1I; see also sex difference in Supplementary Fig. S5; see online supplementary material for a color version of this figure). Alanine (11%), phenylalanine (10%), and histidine (5%) showed modest associations with age. Glutamine was different between the sexes, with a stronger association in males (56%) but a weaker age effect in females (12%), and an overall effect of 34%.

## Temporal trajectories of circulating molecular concentrations

**Figure 2.**
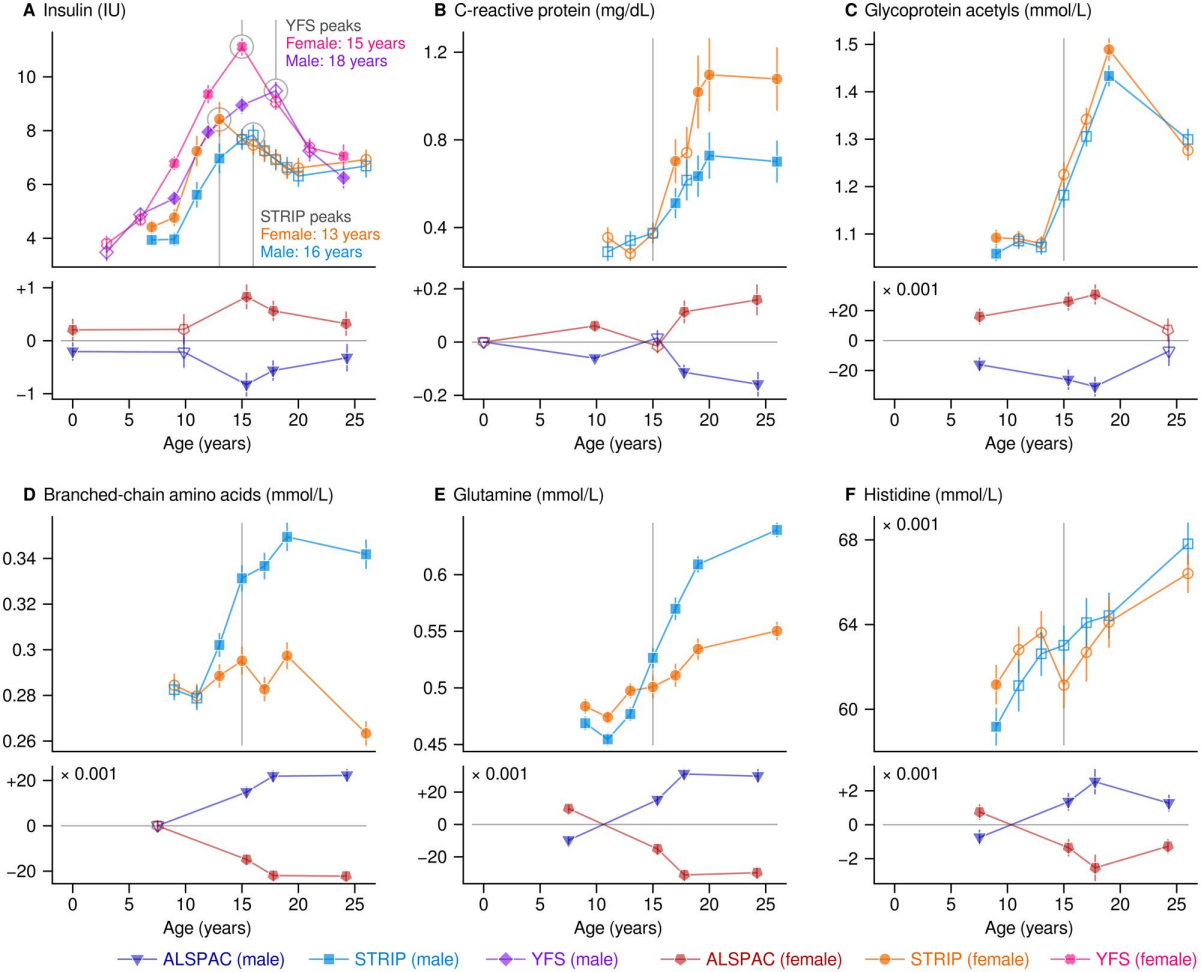
Robust mean values and 95% confidence intervals for selected circulating metabolic measures. The results are calculated separately for each cohort and for males and females. The filled symbols indicate a statistical difference that satisfies the single-test threshold of *P* < 0.0001. The plots consist of two subplots; the lower subplot shows the results from the ALSPAC centered on the mean values of the peer groups. ALSPAC, Avon Longitudinal Study of Parents and Children; IU, insulin unit; STRIP, Special Turku Coronary Risk Factor Intervention Project; YFS, Cardiovascular Risk in Young Finns Study.

Age- and sex-stratified geometric means and 95% confidence intervals for insulin are shown in Fig. 2A and numerical results are provided in Supplementary Table S1 (see online supplementary material for a color version of this table). The highest values were observed between ages 13 and 15 years for girls [max 11.1  insulin units (IU) across cohorts] and between 16 and 18 years for boys (max 9.5 IU); note the difference between the STRIP and the YFS. Females tended to have higher values across ages (median difference 0.42 IU). As shown in Fig. 2B and C, inflammatory markers were also higher in females and increased with age. There was a sex divergence in branched-chain amino acids that resulted in a 22% higher concentration in men at age 24–26 years (Fig. 2D; see also Supplementary Fig. S8; see online supplementary material for a color version of this figure). Glutamine and histidine increased in both sexes; however, the rate was faster in men despite a lower starting point (Fig. 2E and F).

**Figure 3.**
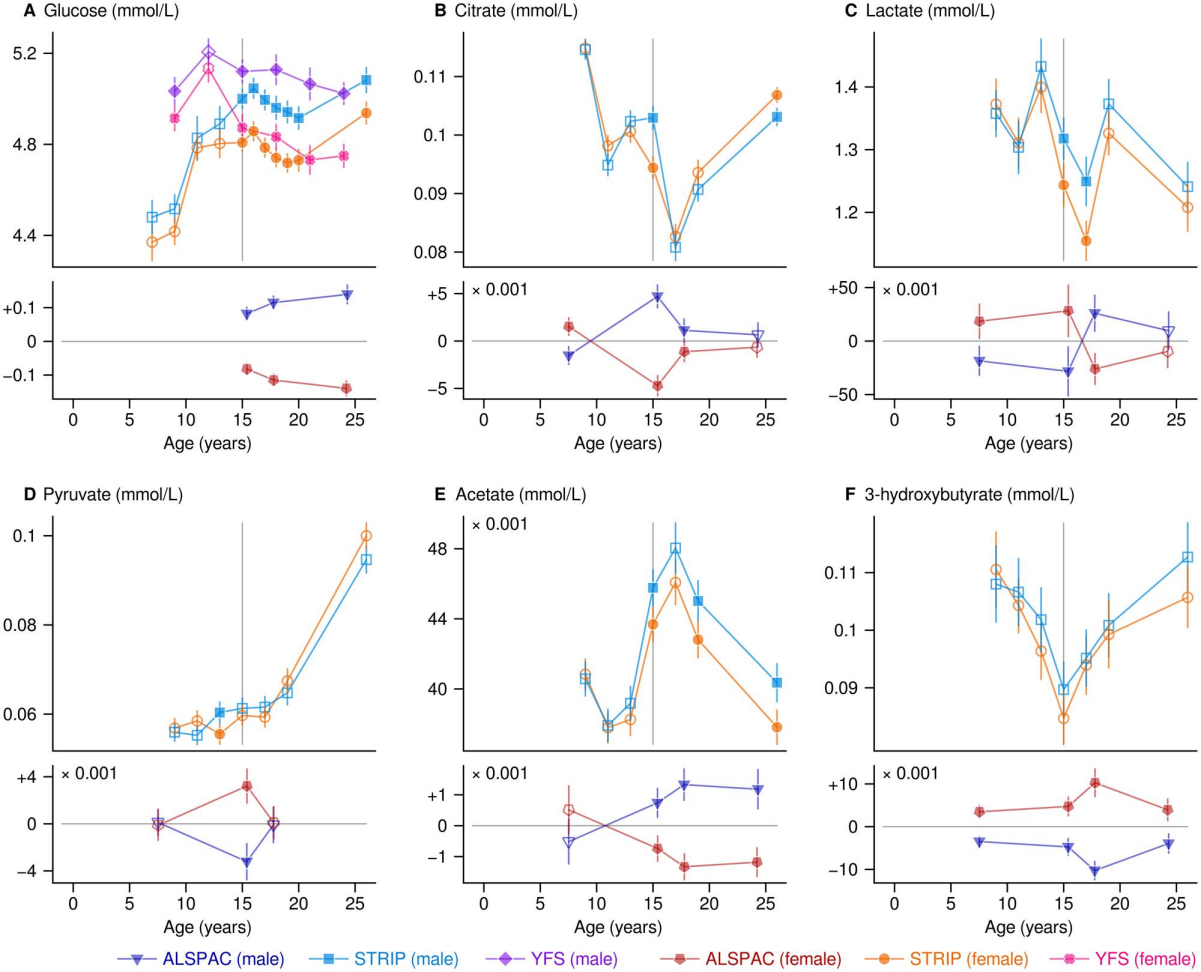
Robust mean values and 95% confidence intervals for selected circulating metabolic measures. The results are calculated separately for each cohort and for males and females. The filled symbols indicate a statistical difference that satisfies the single-test threshold of *P* < 0.0001. The plots consist of two subplots; the lower subplot shows the results from the ALSPAC centered on the mean values of the peer groups. ALSPAC, Avon Longitudinal Study of Parents and Children; STRIP, Special Turku Coronary Risk Factor Intervention Project; YFS, Cardiovascular Risk in Young Finns Study.

Glucose was higher in males (Fig. 3A; median difference 0.19 mmol/L). Citrate followed a u-shaped pattern with a maximal gap of 8.7% between a higher value in boys compared with girls at age 15 years (Fig. 3B). Pyruvate followed a consistent trend upward with age in both sexes (Fig. 3D). Acetate showed no sex difference in children but higher values in adult males compared with females (Fig. 3E; difference of 4.9% at age 24–26 years). The temporal pattern of acetate peaked at age 17 years when citrate was the lowest.

**Figure 4.**
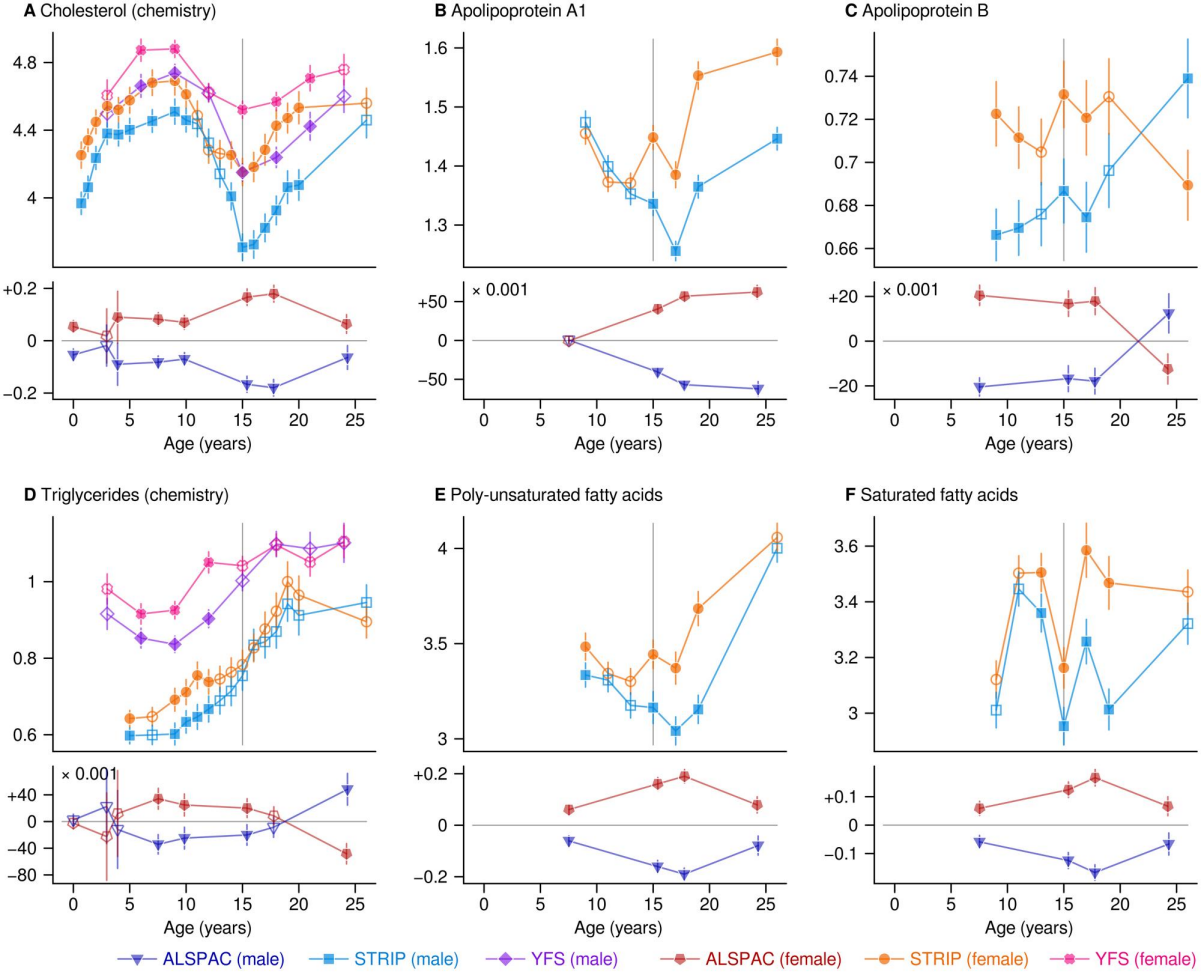
Robust mean values and 95% confidence intervals for selected circulating metabolic measures. The results are calculated separately for each cohort and for males and females. The filled symbols indicate a statistical difference that satisfies the single-test threshold of *P* < 0.0001. The plots consist of two subplots; the lower subplot shows the results from the ALSPAC centered on the mean values of the peer groups. ALSPAC, Avon Longitudinal Study of Parents and Children; STRIP, Special Turku Coronary Risk Factor Intervention Project; YFS, Cardiovascular Risk in Young Finns Study.

Total cholesterol (Fig. 4A) was higher in females (median difference 0.18 mmol/L). A similar undulating trajectory was observed for HDL cholesterol but with a puberty-induced sex divergence (Supplementary Fig. S7; see online supplementary material for a color version of this figure). The same pattern was visible for apolipoprotein A1 (Fig. 4B). Apolipoprotein B was higher in girls (Fig. 4C; median difference 0.041 g/L at age 7–18 years); however, there was a simultaneous increase in young men and a decrease in young women after puberty (lower in females, −0.017 g/L at age 24–26 years). Fatty acids were typically higher in females than in males (Fig. 4E and F, and Supplementary Fig. S9; see online supplementary material for a color version of this figure).

## Emergence of adult male and female metabolic phenotypes

**Figure 5.**
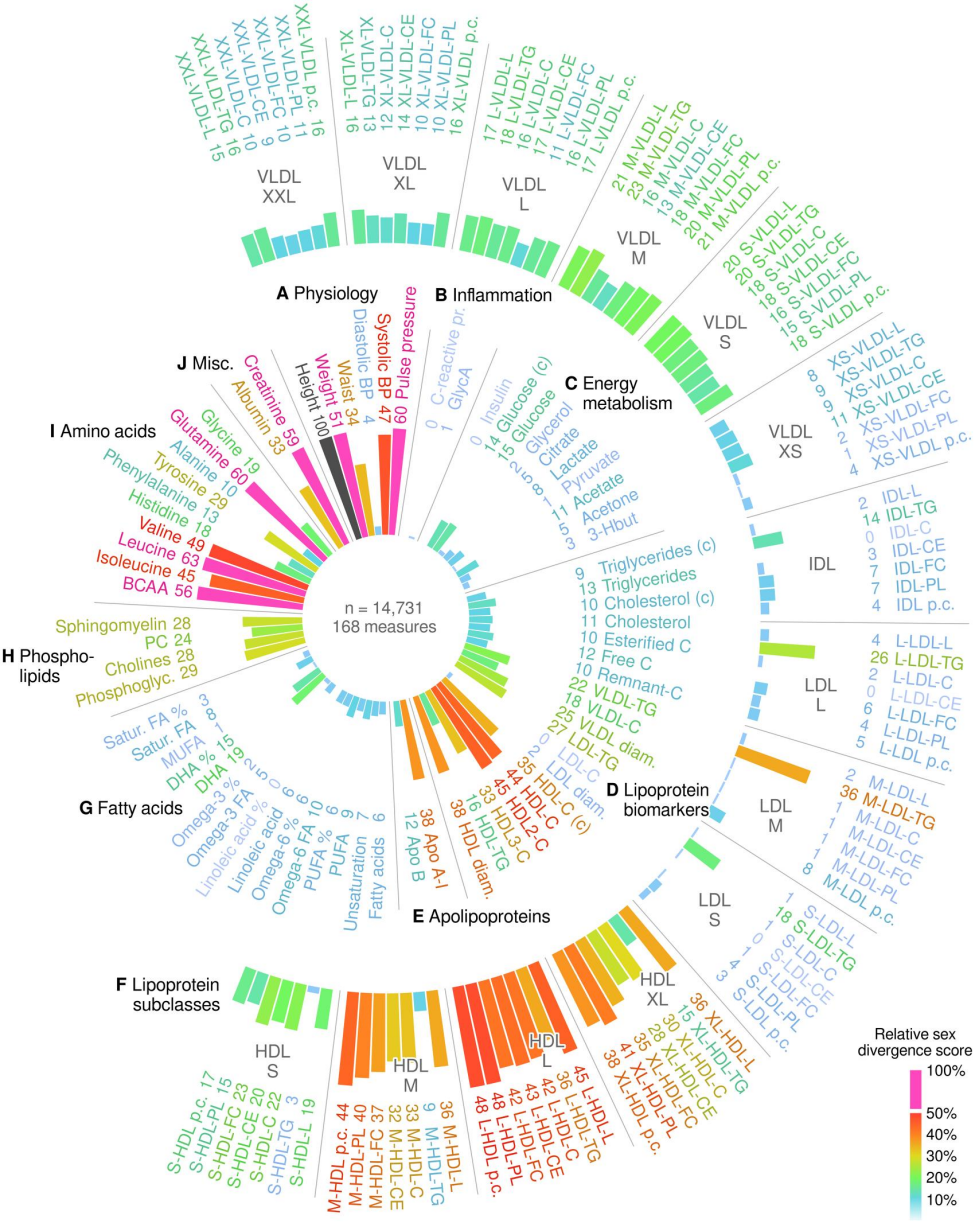
Overview of how sex dimorphism in metabolic measures changed between younger children (age ≤13 years) and young adults (age ≥17 years) in the combined dataset from the STRIP, YFS, and ALSPAC. We defined the sex divergence score Δ_S_ as the absolute difference between the α and β coefficients from the logistic regression modeling of sex differentiation (Δ_S_ = |α − β|, see Methods and Supplementary Figure S10; see online supplementary material for a color version of this figure). A high Δ_S_ indicates that there was a large change in the association between sex and a specific metabolic measure, i.e. puberty affected one sex differently compared with the other. The maximum score was observed for height and it was used as the reference to calculate relative percentages. The exact percentages are written next to the variable names in the figure and the *P*-values are available in Supplementary Table S4 (see online supplementary material for a color version of this figure). The subplot labels refer to the inner circle, except Plot F, which covers the entire outer arc of lipoprotein subclass measures. Apo, apolipoprotein; ALSPAC, Avon Longitudinal Study of Parents and Children; BCAA, branched-chain amino acids; BP, blood pressure; DHA, docosahexaeonic acid; FA, fatty acid; GlycA, glycoprotein acetyls; HDL, high-density lipoprotein; IDL, intermediate-density lipoprotein; LDL, low-density lipoprotein; PUFA, polyunsaturated fatty acids; STRIP, Special Turku Coronary Risk Factor Intervention Project; VLDL, very-low-density lipoprotein; YFS, Cardiovascular Risk in Young Finns Study; lipoprotein subclass lipids: L, total lipid; TG, triglycerides; C, cholesterol; CE, cholesterol esters; PL, phospholipids; pc, particle concentration; lipoprotein subclass sizes: L, large; M, medium; S, small; XS, extra small; XL, extra large; XXL, extremely large.

To eliminate batch effects, we focused on the emergence of sex dimorphism as an alternative indicator of age-dependent metabolic traits. The data from all three study cohorts were divided into two groups by using age cutoffs based on multivariate modeling (Supplementary Fig. S4; see online supplementary material for a color version of this figure). The coefficients for logistic models of male sex are shown in Supplementary Fig. S10 and full numerical results are provided in Supplementary Table S3 (see online supplementary material for color versions of the figure and table). For clarity, we defined the relative sex divergence score (against height) based on the regression results to illustrate associations between puberty and specific metabolic variables (Fig. 5 and Supplementary Table S4; see online supplementary material for a color version of this table). Height (100%), pulse pressure (60%), and creatinine (59%) are classic examples of puberty-induced sex dimorphism (Fig. 5A and H).

Glycoprotein acetyls were not associated with puberty-induced sex divergence (1%, *P *=* *0.35) (Fig. 5B) despite overall lower values in males and an association with age (Fig. 1B). Insulin showed the same temporal stratification pattern without sex divergence (Figs 1C and 5C). Neither glycerol nor pyruvate diverged between sexes during puberty, but were associated with age (Figs 1C and 5C).

HDL cholesterol was age-dependent (Fig. 1D) and substantially lower in males after metabolic puberty transition (35%) (Fig. 5D). Lipoprotein subclass data revealed how IDL and LDL subclasses were the least affected by puberty, except for their triglyceride content (scores between 14% and 36%) whereas large HDL lipids were sensitive indicators of the maturation of sex differences (36–48%) (Fig. 5F). Docosahexaenoic acid (19%), sphingomyelin (28%), and cholines (28%) were also divergent (Fig. 5G and H).

Circulating amino acids were substantially changed between the sexes during puberty (Fig. 5I). Leucine (63%) and glutamine (60%) diverged during puberty to the same degree as creatinine (59%) and body weight (51%), as was hinted at by their strong age-dependence in men (Supplementary Fig. S5; see online supplementary material for a color version of this figure). Other amino acids such as tyrosine—that were less affected by age per se (Fig. 1I)—diverged into higher values in young men compared with women (29%) (see also Supplementary Fig. S10; see online supplementary material for a color version of this figure).

## Discussion

We conducted rigorous analyses of biochemical time series from 14 958 individuals over two decades across three cohorts with careful consideration for batch effects that are inherent to longitudinal datasets. We observed that insulin, glycerol, and glycoprotein acetyls changed with age and were lower in males, but did not exhibit sex divergence during puberty. Substantial sex divergence was observed pre- vs. post-puberty for creatinine, glutamine, HDL subclasses, polyunsaturated fatty acids, and branched-chain amino acids. Neither apolipoprotein B nor LDL cholesterol was associated with age. Overall, metabolic differentiation into adult males and females took place between the ages of 13 and 17 years.

Apolipoprotein B and LDL cholesterol are causal predictors of cardiovascular disease in adults [34–36] and may indicate lifetime risk from birth [4]. Apolipoprotein B appears to be lower in infants and then stable in children [37–39] with some sex divergence towards adulthood [40]. Our longitudinal analyses revealed stable values in girls aged 9–17 years that then flipped into lower values in adult females vs. males; however, temporal changes were small compared with differences between individuals. Based on our new large-scale results on lipoprotein subclasses, we speculate that the sex flip reflects both a sex-specific increase in apolipoprotein B numbers that come from VLDL particles (VLDL particle concentrations increase faster in males throughout childhood) and changes in LDL metabolism between adult men and women (Fig. 5F and Supplementary Figs S10 and S11; see online supplementary material for color versions of these figures). From a practical point of view, LDL cholesterol is also stable and therefore additional information from apolipoprotein B may be limited. Nevertheless, these new results across multiple cohorts and previous tracking analyses [41] support apolipoprotein B as an early indicator of lifelong cardiovascular risk.

Insulin resistance increases in puberty and manifests as higher circulating insulin [3, 42]. Our data indicate that the insulin peak occurred earlier in girls compared with boys, and earlier in the STRIP (launched in 1990) compared with the YFS (born before 1980). These observations are compatible with the historical trend of earlier puberty in younger generations [43] and they imply that insulin resistance in adolescence may be too variable to work as a robust predictive risk factor. We also saw a dip in 3-hydroxybutyrate at age 15 years when insulin resistance was the highest, which is the opposite of what happens in adults [44]. Acetate—another key molecule in energy metabolism [45] that may increase insulin sensitivity [46]—peaked at age 17 years, possibly indicating the culmination of biomass accumulation towards the end of the growth spurt.

Increased lipolysis from excess adiposity promotes glycerol release into circulation in obese adolescents [47] and in adults [48]. Our new results show that insulin increased without any change in glycerol at age 13 years (no association) followed by lower insulin and higher glycerol at age 17 years (adult-like association). This complex pattern suggests that the regulatory mechanisms between insulin and lipolysis may be different in children compared with adults, possibly due to the energy demands from growing.

Citrate metabolism is important for adult health [49] but less is known about citrate in children. A previous metabolomics study reported lower concentrations in boys at age 12 years [7]. We observed higher serum concentrations in boys at age 15 years whereas another study found lower urinary citrate excretion in boys of the same age [50]. It is possible that the higher need for citrate for bone formation during the male growth spurt may explain the higher availability in blood [51].

Elevated branched-chain amino acids in adults are risk factors for cardiometabolic diseases such as type 2 diabetes [18, 52] and differences between adults and children have been observed [7]. Our data show that amino acids (especially leucine and glutamine) are among the strongest indicators of metabolic maturation into adult sex. Notably, isoleucine and leucine returned to prepubertal concentrations in females after the insulin peak whereas the pubertal increase became permanent in males. This, together with the lack of sex difference in adult insulin, suggests that the regulatory network between insulin and amino acids was rewired to a greater extent in males compared with females. We speculate that the rewiring reflects the need to maintain the higher muscle-to-fat ratio in men and may contribute to sex differences in the epidemiology of insulin resistance [53].

In adults, elevated glycoprotein acetyls indicate chronic inflammation [20] and are associated with a wide variety of diseases [22]. Our study revealed that glycoprotein acetyls were stable before puberty (higher in girls), then increased by 40%, and then remained elevated in adults. C-reactive protein behaved similarly, as expected [54]; however, C-reactive protein was higher in adult females (possibly due to contraception [55, 56] or menstruation [57]) whereas the sex difference in glycoprotein acetyls disappeared after puberty. These temporal associations are substantial and may require age- and sex-specific reference ranges if glycoprotein acetyls are incorporated into metabolic assessments of children and adolescents.

The strengths of this study include high statistical power, multiple cohorts, and a long follow-up from early childhood to young adults. Furthermore, our results replicate earlier findings for those biomarkers for which longitudinal studies were available [2, 5, 54, 58]. The study is limited by the young age of the participants that prevents direct analyses of incident disease end points. We caution against extrapolating the results into other ethnic groups and there are inherent technical challenges of long-term follow-up studies that may reduce the accuracy of the results. Nevertheless, the overall picture is clear: most metabolic traits exhibited meaningful associations with the growth and development of children, including emerging cardiometabolic risk factors such as branched-chain amino acids and glycoprotein acetyls. Conversely, apolipoprotein B and LDL cholesterol were stable, which indicates their potential utility for early assessments of lifetime cardiometabolic risk.

## Ethics approval

This study did not involve recruitment of study subjects or new biomedical experiments. ALSPAC: ethical approval for the study was obtained from the ALSPAC Ethics and Law Committee and the Local Research Ethics Committees. The STRIP was approved by the associated university and hospital district ethical authorities [24]. The YFS was approved by the ethical committees of five Finnish universities with medical schools (Helsinki, Kuopio, Oulu, Tampere and Turku [26]). ALSPAC: consent for biological samples has been collected in accordance with the Human Tissue Act (2004); informed consent for the use of data collected via questionnaires and clinics was obtained from participants following the recommendations of the ALSPAC Ethics and Law Committee at the time [28]. STRIP: written informed consent was obtained from parents at study entry and from the participants at ages 15, 18, and 26 years. YFS: all participants gave written informed consent. This study reports only summary statistics. No information from a single person is published.

## Supplementary Material

dyaf026_Supplementary_Data

## Data Availability

The ALSPAC data are available to researchers via application (URL: https://www.bristol.ac.uk/alspac/). This study was approved under the project B3830. The Finnish datasets used in the current study are available from the cohorts through the application process for researchers who meet the criteria for access to confidential data (URL: http://youngfinnsstudy.utu.fi and URL: https://stripstudy.utu.fi/). Regarding these data, the ethics committee has concluded that, under applicable law, the data from these studies cannot be stored in public repositories or otherwise made publicly available. The data controller may permit access on a case-by-case basis for scientific research, not, however, to individual participant level data, but to aggregated statistical data, which cannot be traced back to data for the individual participants.
