# Supplementary material for: Metabolic transition from childhood to adulthood based on two decades of biochemical time series in three longitudinal cohorts
Source: Int J Epidemiol. 2025 Mar 26;54(2):dyaf026. doi: 10.1093/ije/dyaf026 (PMC11947525; doi:10.1093/ije/dyaf026)
Supplement: dyaf026_Supplementary_Data [file dyaf026_supplementary_data.zip › ije-2024-05-0625-File008.docx]

# **Supplementary figures**

S1 Age coverage of the biochemical data

S2 Illustration of systematic differences in clinical lipids between cohorts

S3 Residual plots of non-linear age trajectory models for metabolic traits

S4 Emergence of adult metabolic sex differentiation during puberty

S5 Associations between age and circulating metabolic measures

S6 Temporal trajectories for metabolic measures that were responsive to the health
intervention in the Special Turku Coronary Risk Factor Intervention Project

S7 Age-specific mean values for basic physiological and biochemical measures

S8 Age-specific mean values for circulating amino acids

S9 Age-specific mean values for circulating lipids

S10 Sex differences before and after puberty transition

S11 Age-specific mean values for atherogenic lipoprotein fractions

**
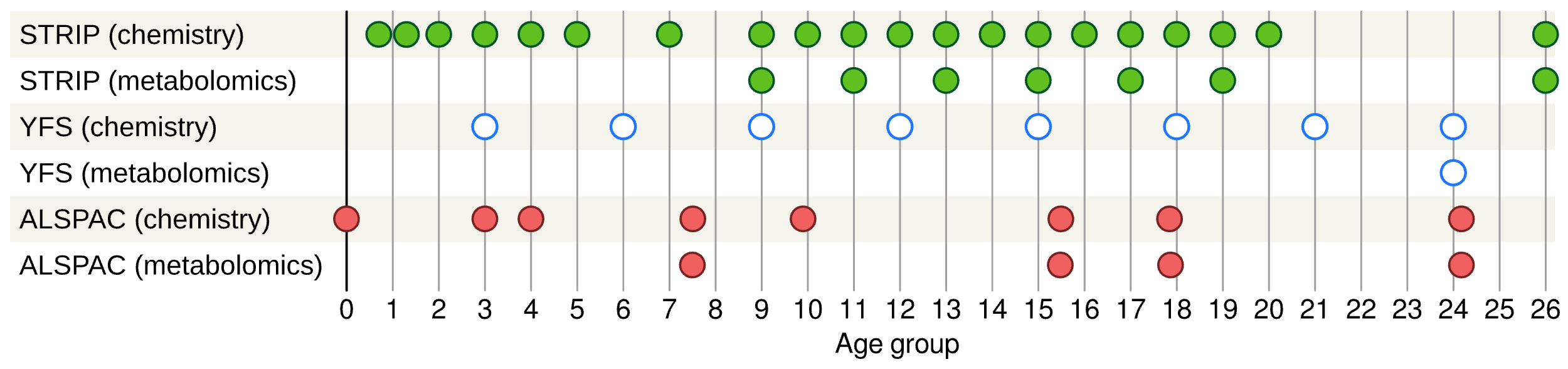
**

**Supplementary Figure S1:** Age coverage of the biochemical data used in this study. For clarity, mean age for each visit is shown for ALSPAC. Given the longitudinal structure, not all participants made all study visits and not all individuals had all the variables available at specific visits, therefore a single number for the study size may be misleading. For this reason, we first defined the maximal study size according to the availability of any biochemical measurement that was used in any of the statistical results included in the study (please see Table 1). Secondly, we listed in Methods the minimum and maximum numbers per visit of ALSPAC and STRIP participants with usable data to more accurately summarize the sparse nature of the dataset. Thirdly, the exact numbers of participants that contributed biochemical data to specific analyses are reported alongside the respective results (please see Supplementary Tables S1-S4). Abbreviations: ALSPAC (Avon Longitudinal Study of Parents and Children), STRIP (Special Turku Coronary Risk Factor Intervention Project) and YFS (Cardiovascular Risk in Young Finns Study).

**
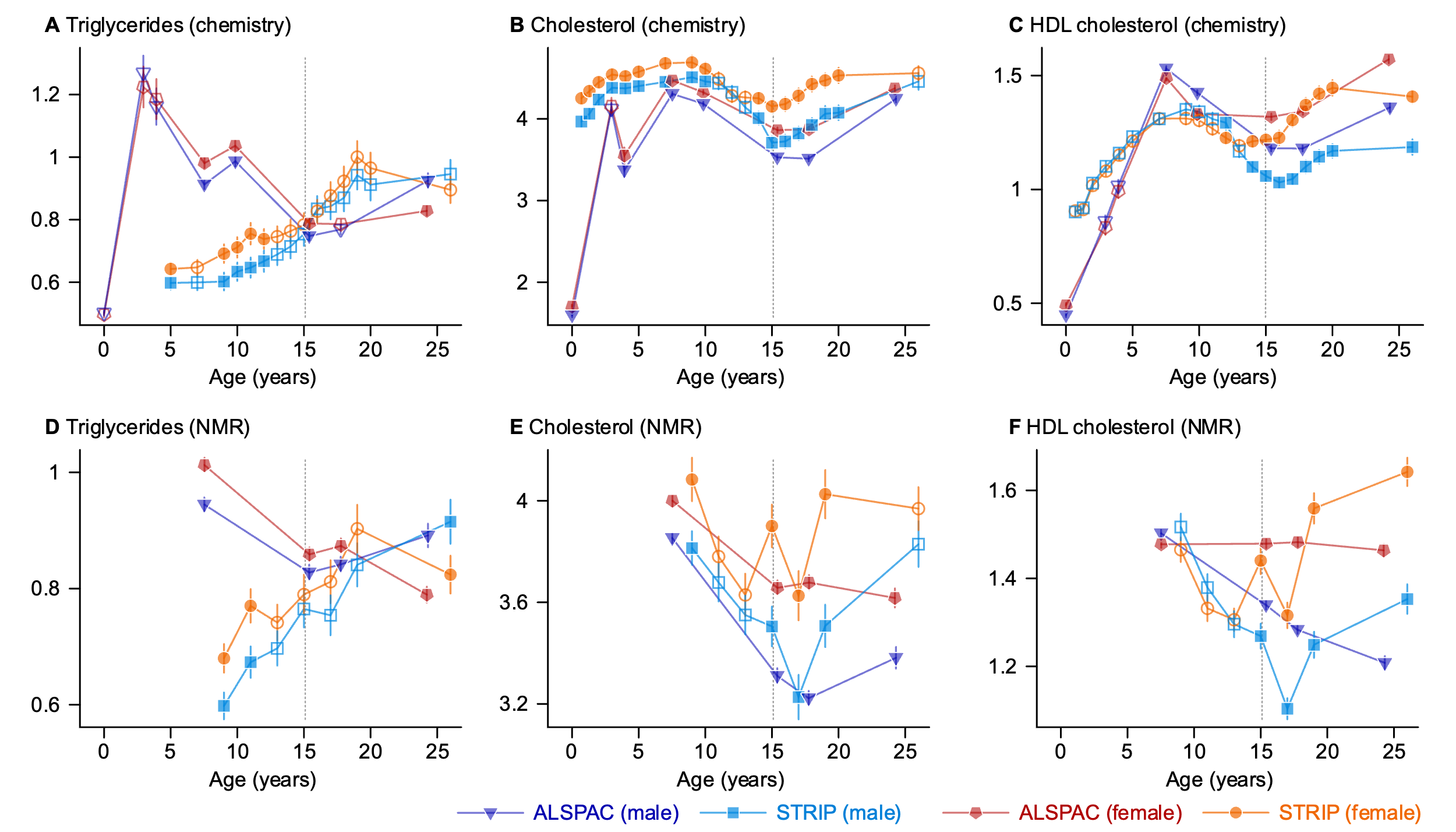
**

**Supplementary Figure S2:** Illustration of systematic differences between the STRIP and the ALSPAC cohorts (YFS was similar to STRIP and omitted to improve visual clarity). These differences were not explained by the measurement technology as both the standard biochemical assay for triglycerides (Plot A) and the corresponding NMR measure (Plot D) were substantially higher in ALSPAC below age 10. The measurements at age 0 were from cord blood. Abbreviations: ALSPAC (Avon Longitudinal Study of Parents and Children), HDL (high-density lipoprotein), NMR (nuclear magnetic resonance) and STRIP (Special Turku Coronary Risk Factor Intervention Project).

**
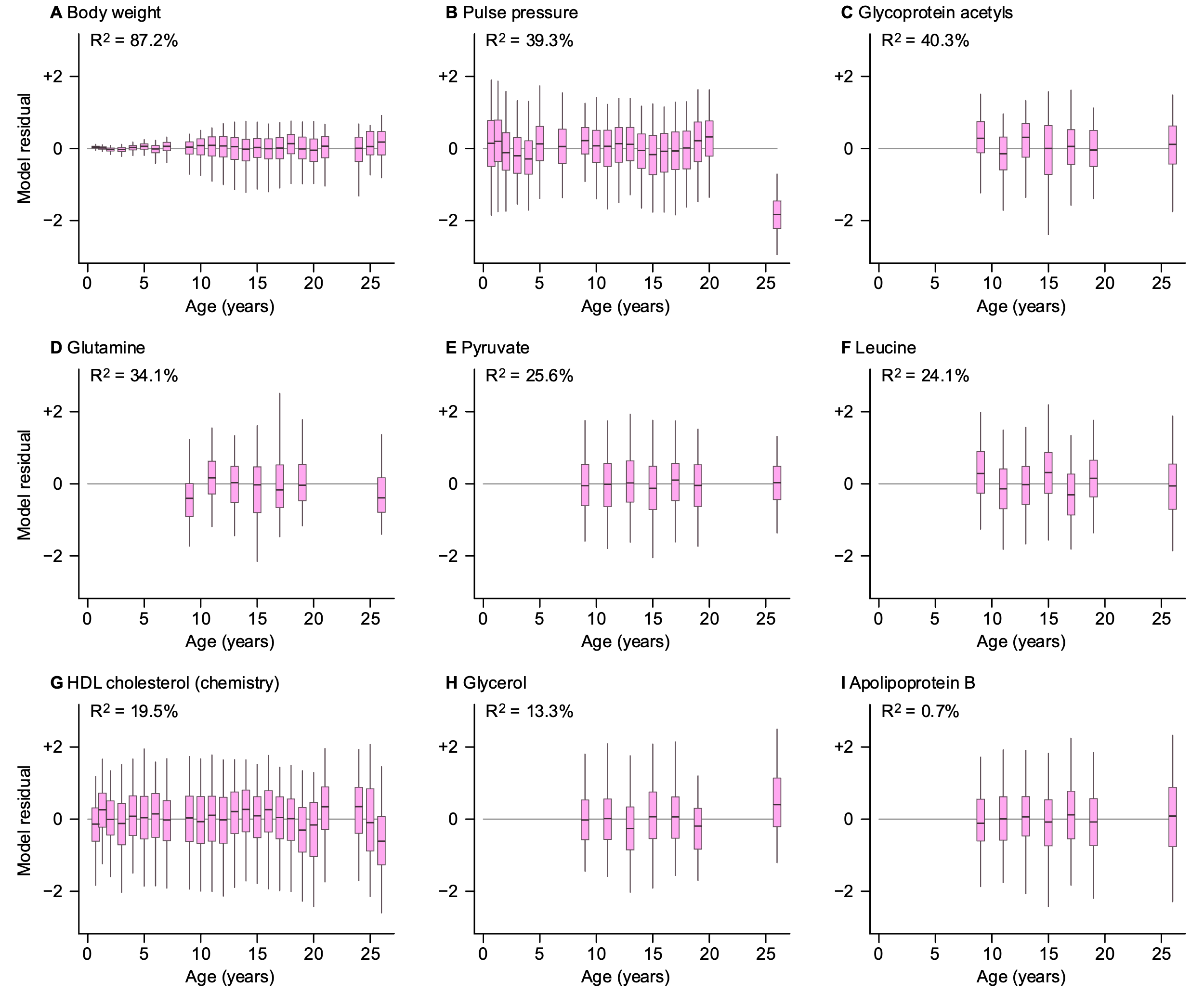
**

**Supplementary Figure S3:** Selected examples of fitting non-linear trajectory models to metabolic traits, sorted approximately from high to low R^2^ value. The median is indicated in the middle, the coloured box shows the inter-quartile range and the vertical lines show the 95% range. Good model fit was prioritised for biochemical traits (not for physiology) as they represent novel data in this study. **A**) Body weight and height (not shown here) are examples of heteroscedastic data that the automatic preprocessing did not recognize as having a skewed distribution. Note also how the overall narrow residual distributions reflect the high proportion of variance that was removed by age. **B**) Pulse pressure is an example where the mitigation of outlying batches and the relatively large gap to the last time point creates a partially biased model. **C–I**) Residual distributions for biochemical traits indicated satisfactory model fits across a variety of molecules and R^2^ values. We did not observe heteroscedastic residuals nor extreme outlier visits that caused unacceptable bias.

We used the cumulative Student’s t-distribution as the basis for sigmoidal components that were added together according to the coefficients of the fitted linear regression model. This choice was motivated by the extra stability sigmoidal shapes provided at the ends of the age range. The number and scale of the shapes were automatically determined based on the number of available time points. To reduce outsized impact from outlying time points (or from overfitting), we incorporated a leave-batch-out cross-validation scheme for calculating a conservative estimate for the amount of variance explained by age. For example, with seven time points with NMR data, the procedure started with excluding all but five samples from the earliest visit (without the five, some of the fits would fail completely). The aforementioned curvilinear regression model was then fitted to the remaining data and all the values not used for training were predicted and stored as validation results. Next, the same procedure was done for the second time point and so on until the seventh visit. The final R^2^ result against observed concentrations was calculated from the predicted values that were set aside as testing samples. This cross-validation scheme was nested within a bootstrapping framework that provided replicate rounds and estimates for the confidence intervals of R^2^.

Because we were fitting curves to a large number of traits, manually tailoring models for each trait would have been at risk of cherry picking suitable parameterizations based on data thus ignoring the multiple testing effect that some trajectories may have been better suited to a particular model type by chance (e.g. some trajectories appeared linear, some were quadratic and some even sinusoidal). Subsequently, we relied on a pre-defined automated pipeline that was optimized for all traits in parallel but without manual tuning for any specific metabolic variable.

Abbreviations: HDL (high-density lipoprotein).

**
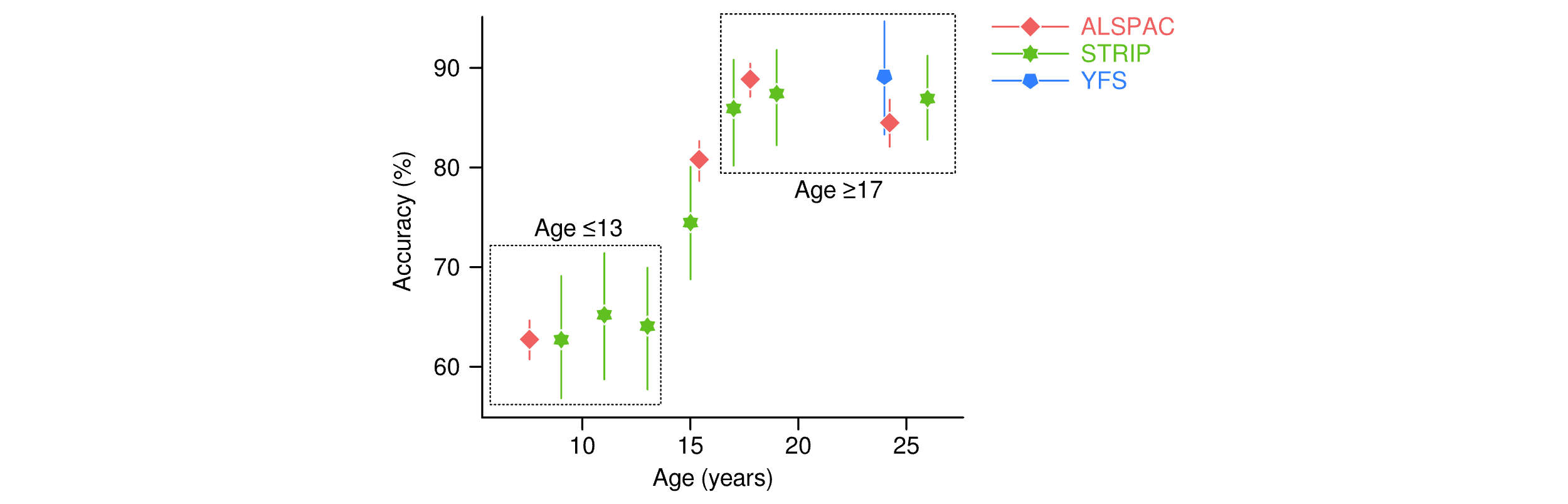
**

**Supplementary Figure S4:** We used a machine learning model to determine how different boys and girls were at different ages. This is a classifier that predicts whether a person is male or female based on their metabolic profile. By training a dedicated classifier using data from, say, age seven only, we can assess how differentiated boys and girls are at that age. The classification accuracy, expressed as the percentage of correct predictions, thus measures how much metabolic sex dimorphism exists within a given peer group.

In the plot, all the markers from 7-13 years of age are within 60-70% accuracy and all the markers from 17-26 years are within 80-90%. From these two stable regions we concluded that the gap in between is the transition period, indeed, the markers around 15 years land between the younger and older accuracy levels. Consequently, we consider the differentiation into adult sex-specific metabolism to start from 13 and end at 17 on average.

Projections to latent structures (PLS) was employed to classify participants into male or female based solely on 162 biochemical variables (physiological traits were excluded). Prediction accuracy was defined as the frequency of correct classification for an adjusted setting with exactly the same number of males and females. A separate PLS model was constructed for each age group and cohort. First, the data were pre-processed and standardized as described in Methods. PLS models that had between 2 and 20 components were then fitted within the bootstrapping framework as described in Methods. Within a bootstrapping round, approximately a third of samples were excluded from training due to random sampling with replacement. The performance of the model was evaluated by predicting the sex for these testing samples. The number of PLS components that produced the best result was chosen as the final model complexity parameter. We observed that sex differences were stable until age 13, then the model accuracy jumped, before stabilizing again after 17.

Abbreviations: ALSPAC (Avon Longitudinal Study of Parents and Children), STRIP (Special Turku Coronary Risk Factor Intervention Project) and YFS (Cardiovascular Risk in Young Finns Study).

**
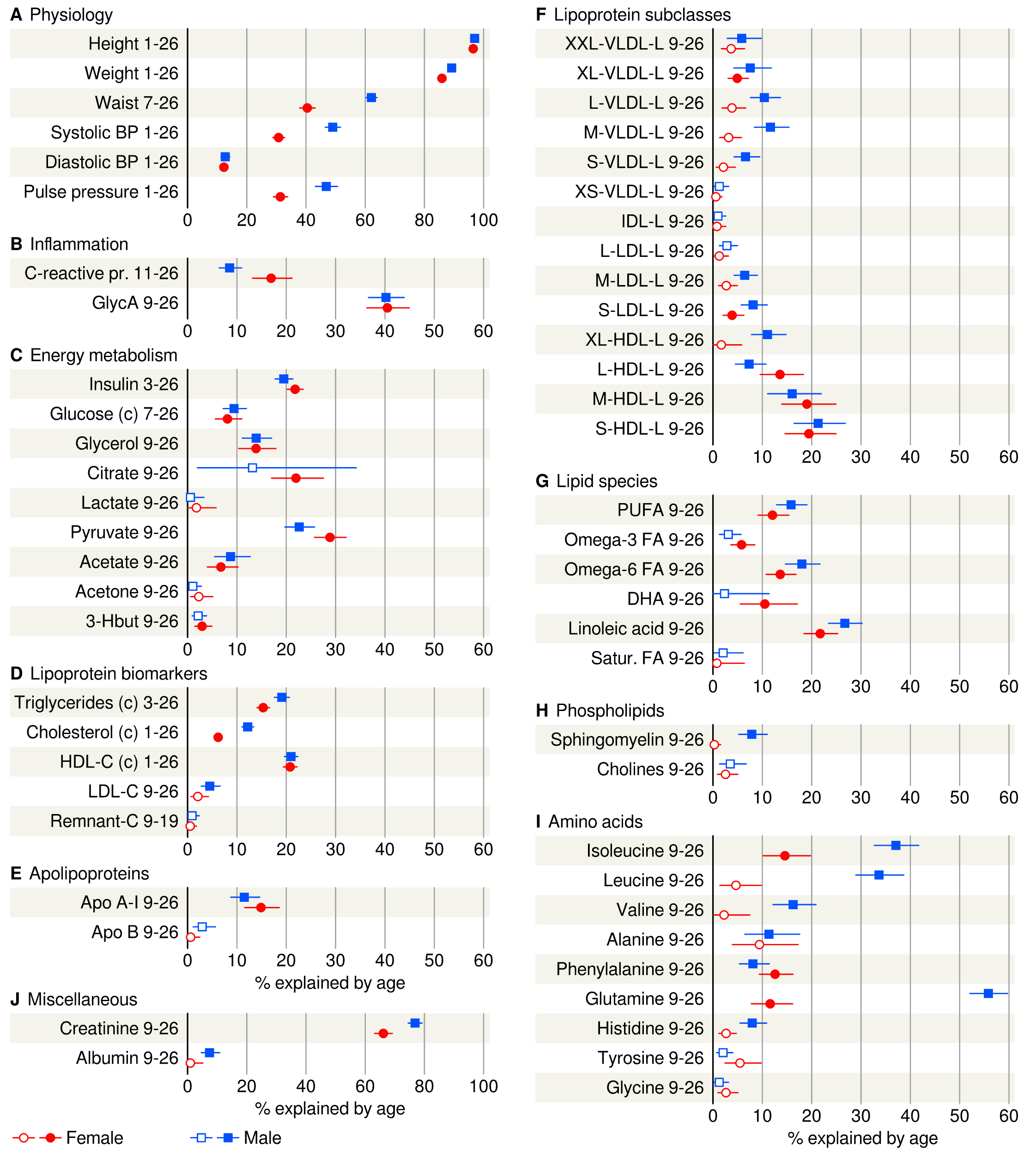
**

**Supplementary Figure S5:** Associations between age and circulating metabolic measures, depicted as % variance explained (R^2^ with 95% confidence intervals) by a curvilinear regression model of age. Results for height, weight, waist, C-reactive protein, insulin, glucose and clinical lipids were calculated from STRIP and YFS datasets. Other results come from STRIP only. The data coverage with respect to age range is written next to the variable name. Filled symbols indicate R^2^ values greater than zero that satisfied the threshold *P* < 0.0001. Abbreviations: Apo (apolipoprotein), BP (blood pressure), DHA (docosahexaeonic acid), FA (fatty acid), GlycA (glycoprotein acetyls), HDL (high-density lipoprotein), IDL (intermediate-density lipoprotein), LDL (low-density lipoprotein), PUFA (polyunsaturated fatty acids), STRIP (Special Turku Coronary Risk Factor Intervention Project), VLDL (very-low-density lipoprotein) and YFS (Cardiovascular Risk in Young Finns Study). Lipoprotein subclass sizes denoted by extra small (XS) to extremely large (XXL). Total subclass lipids denoted as ‘L’ and cholesterol as ‘C’.

**
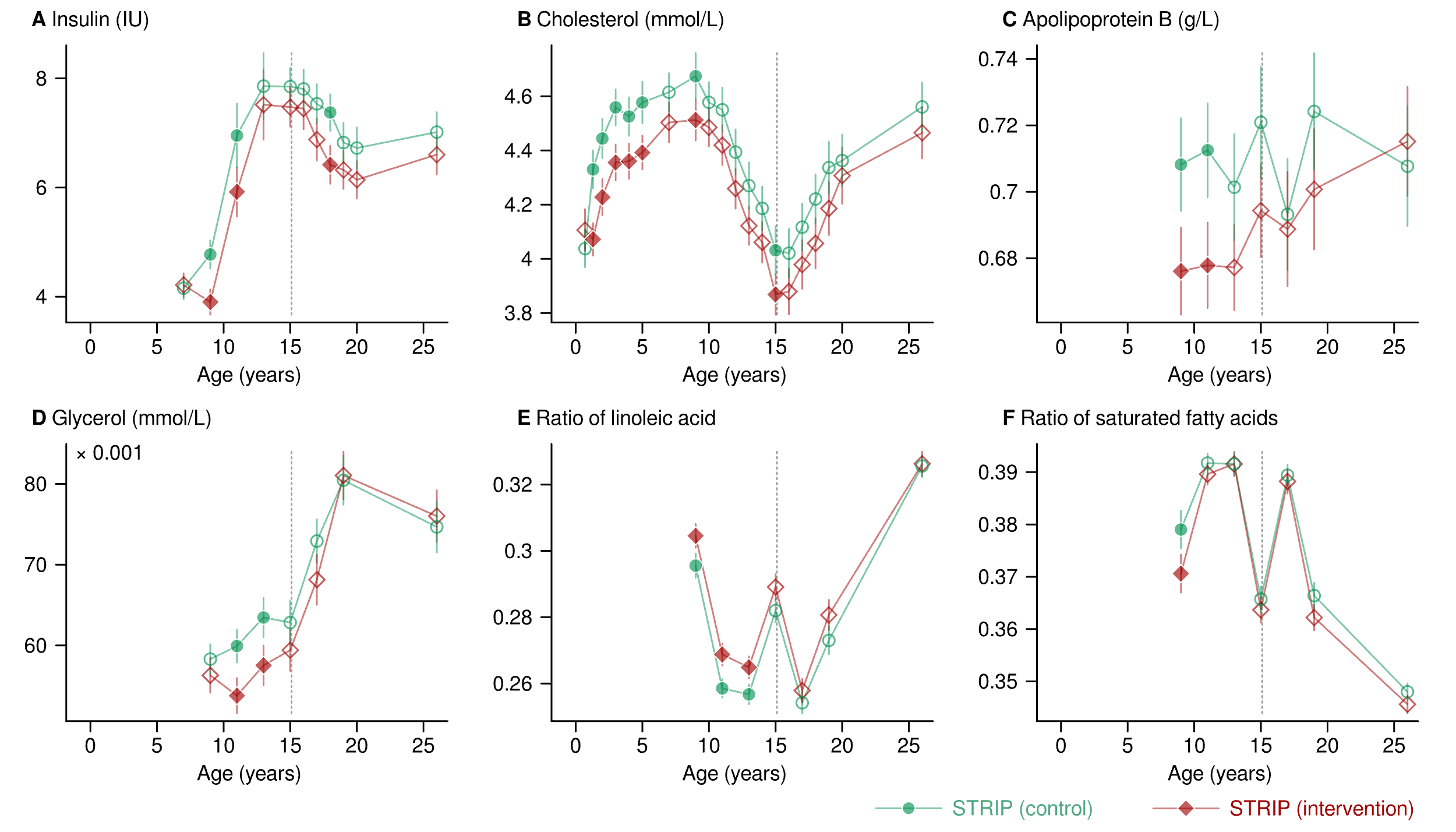
**

**Supplementary Figure S6:** Examples of temporal trajectories for metabolic measures that were responsive to the health intervention in the STRIP trial. The intervention started from first year and ended at age 20. Robust means and 95% confidence intervals are depicted. The filled symbols indicate a statistical difference that satisfies the single-test threshold *P* < 0.0001. Abbreviations: STRIP (Special Turku Coronary Risk Factor Intervention Project).

**
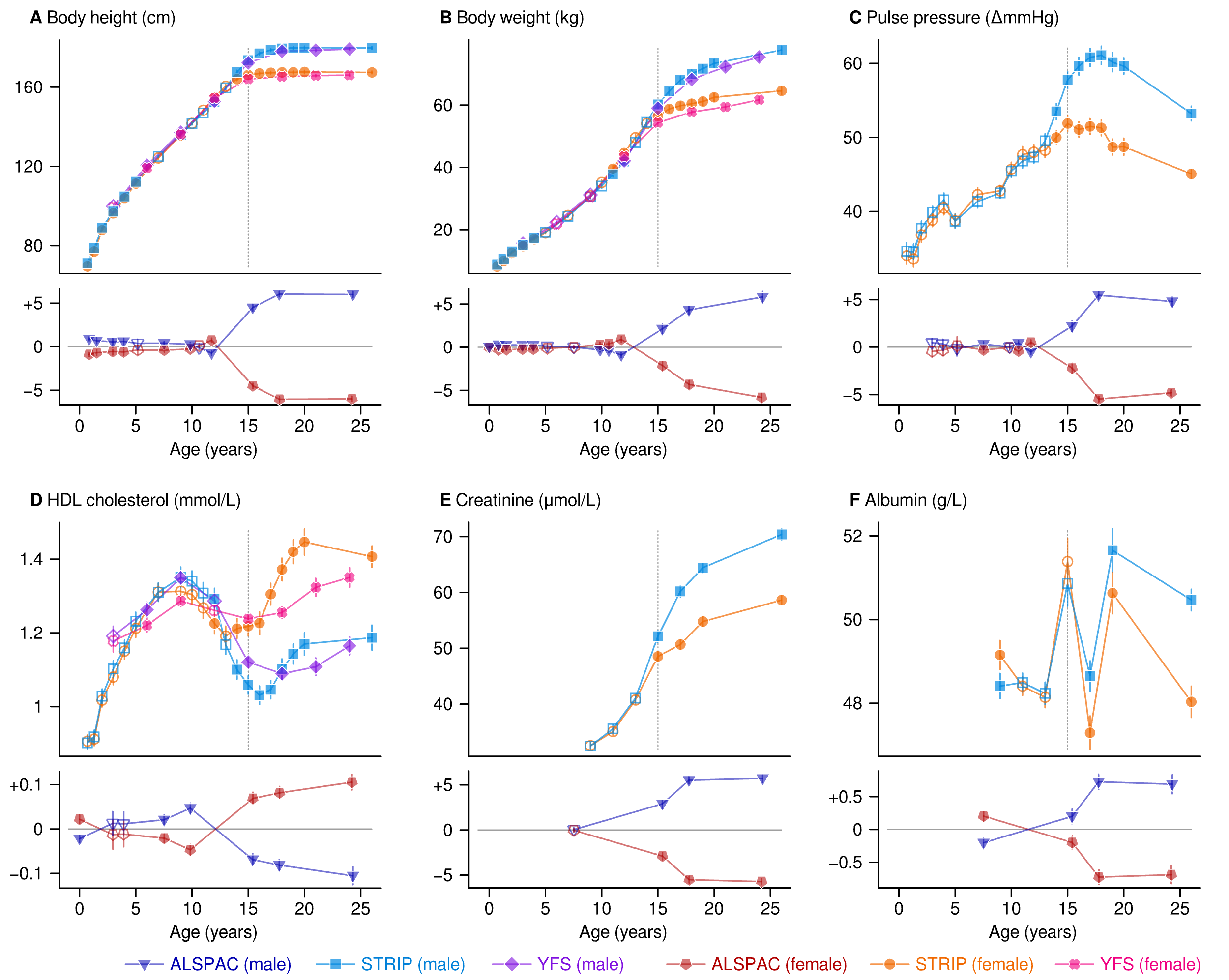
**

**Supplementary Figure S7:** Robust mean values and 95% confidence intervals for selected circulating metabolic measures. The results are calculated separately for each cohort and for males and females. The filled symbols indicate a statistical difference that satisfies the single-test threshold *P* < 0.0001. The plots consist of two subplots; the lower subplot shows the results from ALSPAC centred around the mean values of the peer. Abbreviations: ALSPAC (Avon Longitudinal Study of Parents and Children), HDL (high-density lipoprotein), STRIP (Special Turku Coronary Risk Factor Intervention Project) and YFS (Cardiovascular Risk in Young Finns Study).

**
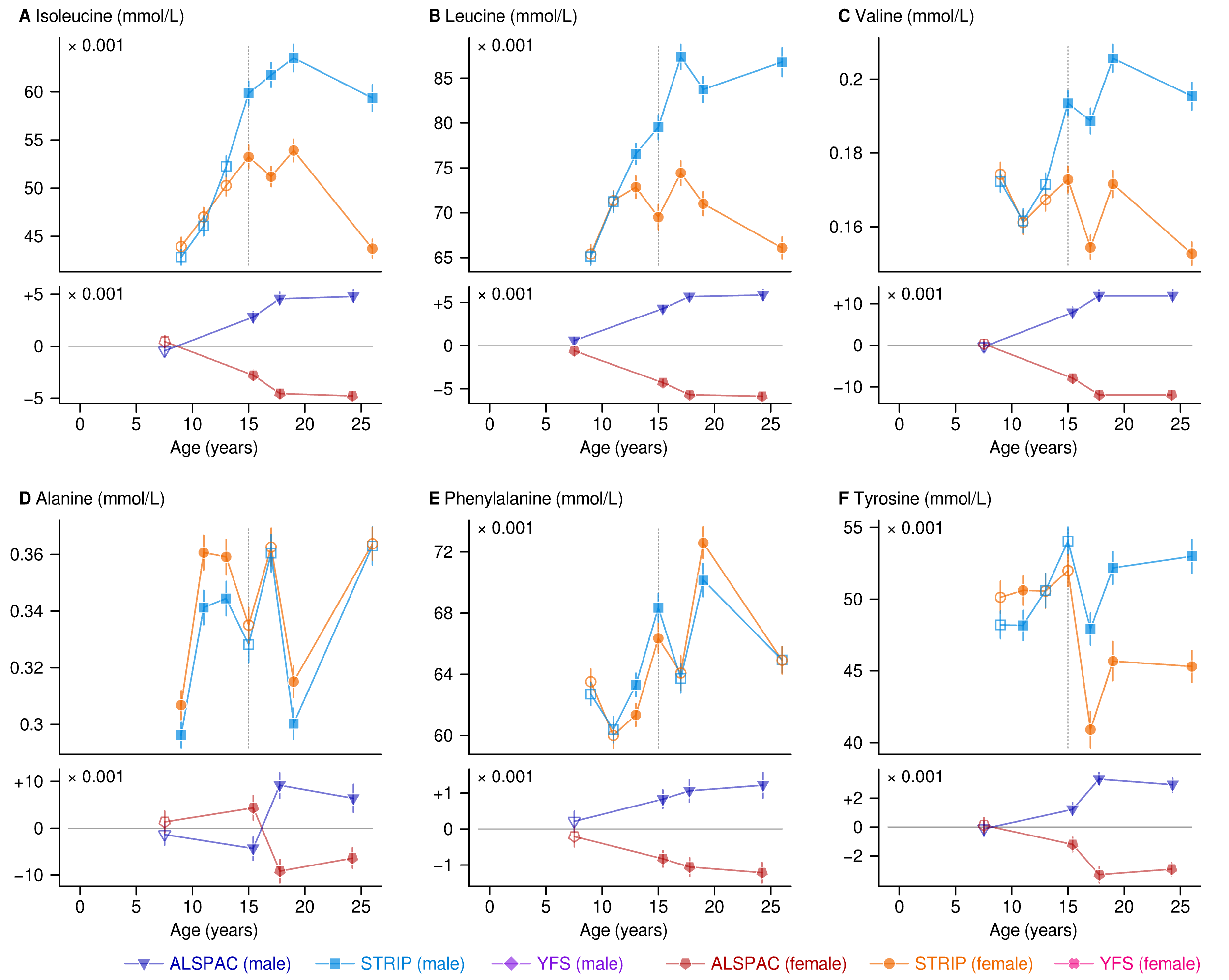
**

**Supplementary Figure S8:** Robust mean values and 95% confidence intervals for selected circulating metabolic measures. The results are calculated separately for each cohort and for males and females. The filled symbols indicate a statistical difference that satisfies the single-test threshold P < 0.0001. The plots consist of two subplots; the lower subplot shows the results from ALSPAC centred around the mean values of the peer groups. Abbreviations: ALSPAC (Avon Longitudinal Study of Parents and Children), STRIP (Special Turku Coronary Risk Factor Intervention Project) and YFS (Cardiovascular Risk in Young Finns Study).

**
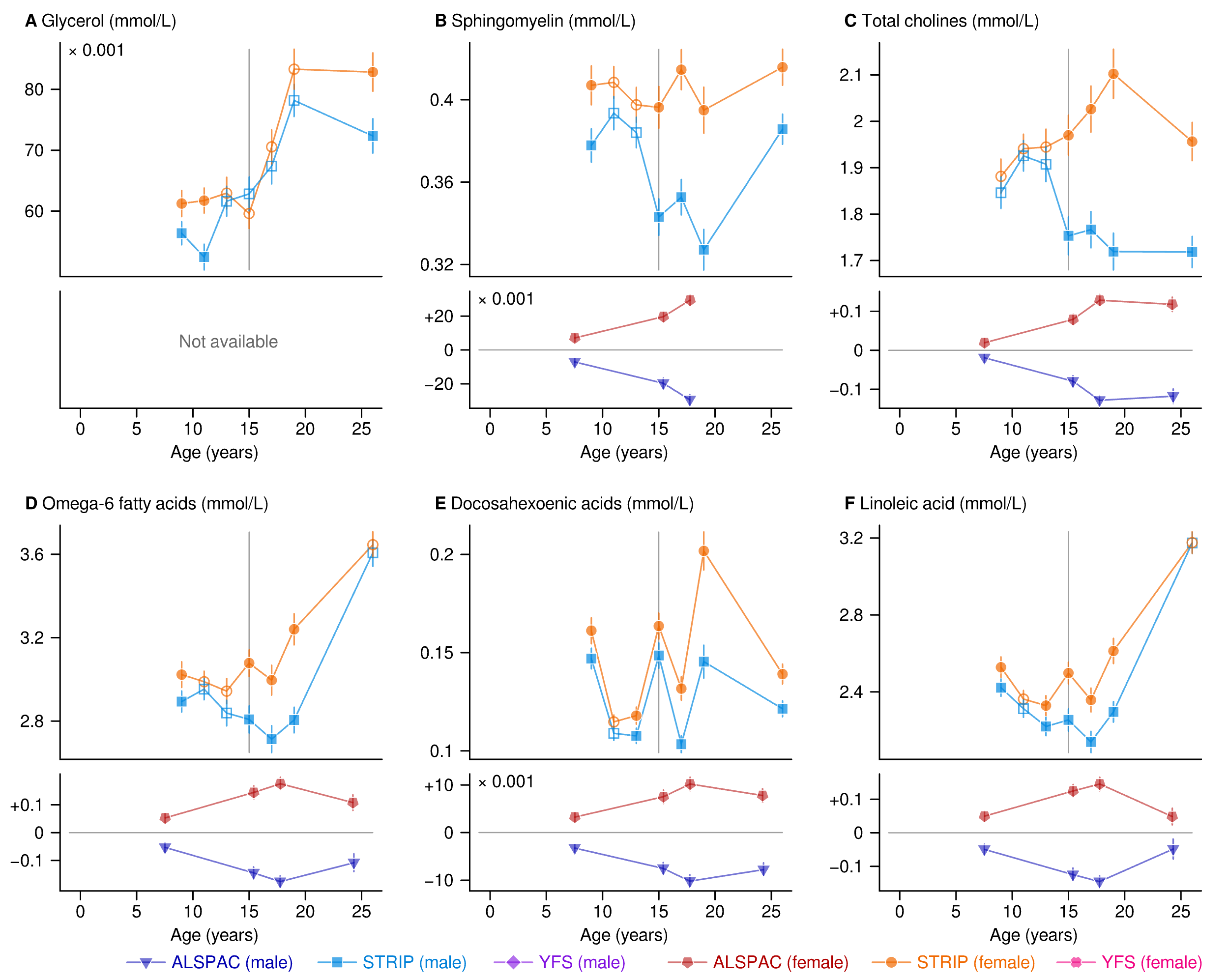
**

**Supplementary Figure S9:** Robust mean values and 95% confidence intervals for selected circulating metabolic measures. The results are calculated separately for each cohort and for males and females. The filled symbols indicate a statistical difference that satisfies the single-test threshold P < 0.0001. The plots consist of two subplots; the lower subplot shows the results from ALSPAC centred around the mean values of the peer groups. Abbreviations: ALSPAC (Avon Longitudinal Study of Parents and Children), STRIP (Special Turku Coronary Risk Factor Intervention Project) and YFS (Cardiovascular Risk in Young Finns Study).

**
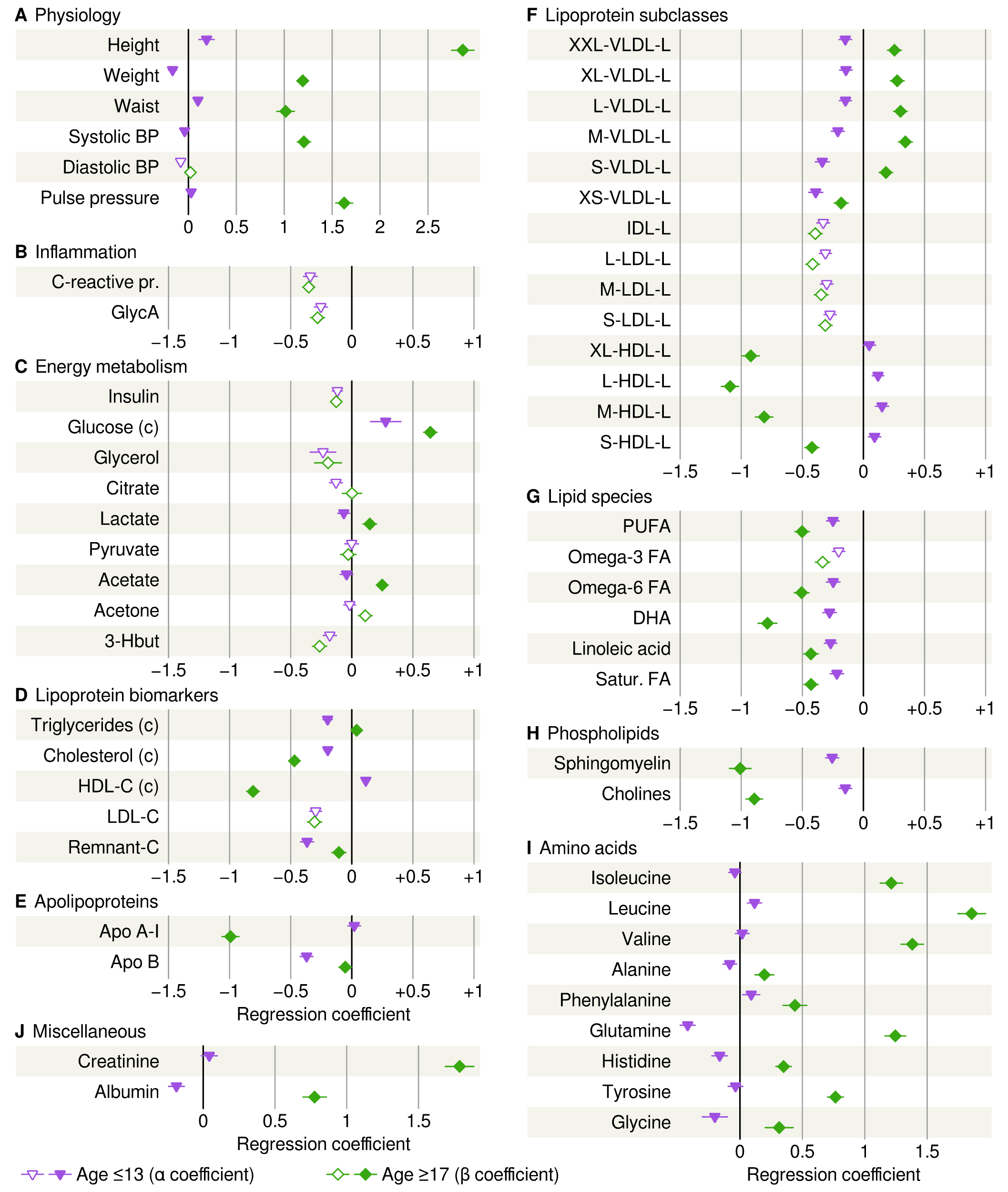
**

**Supplementary Figure S10:** Comparison of metabolic sex differences before and after puberty-induced metabolic transition in the combined dataset from STRIP, YFS and ALSPAC. The symbols show the regression coefficients and 95% confidence intervals from two sets of logistic regression models of the male sex; the first set includes data points at ages up to 13 (α coefficient), and the second includes data points starting from age 17 (β coefficient). A negative coefficient indicates a higher molecular concentration in females, and a positive coefficient indicates a higher value in males. Filled symbols indicate a deviation between pre- and post-pubertal coefficient that satisfies the threshold P < 0.0001. Abbreviations: Apo (apolipoprotein), BP (blood pressure), DHA (docosahexaeonic acid), FA (fatty acid), GlycA (glycoprotein acetyls), HDL (high-density lipoprotein), IDL (intermediate-density lipoprotein), LDL (low-density lipoprotein), PUFA (polyunsaturated fatty acids), STRIP (Special Turku Coronary Risk Factor Intervention Project), VLDL (very-low-density lipoprotein) and YFS (Cardiovascular Risk in Young Finns Study). Lipoprotein subclass sizes denoted by extra small (XS) to extremely large (XXL). Total subclass lipids denoted as ‘L’ and cholesterol as ‘C’.

**
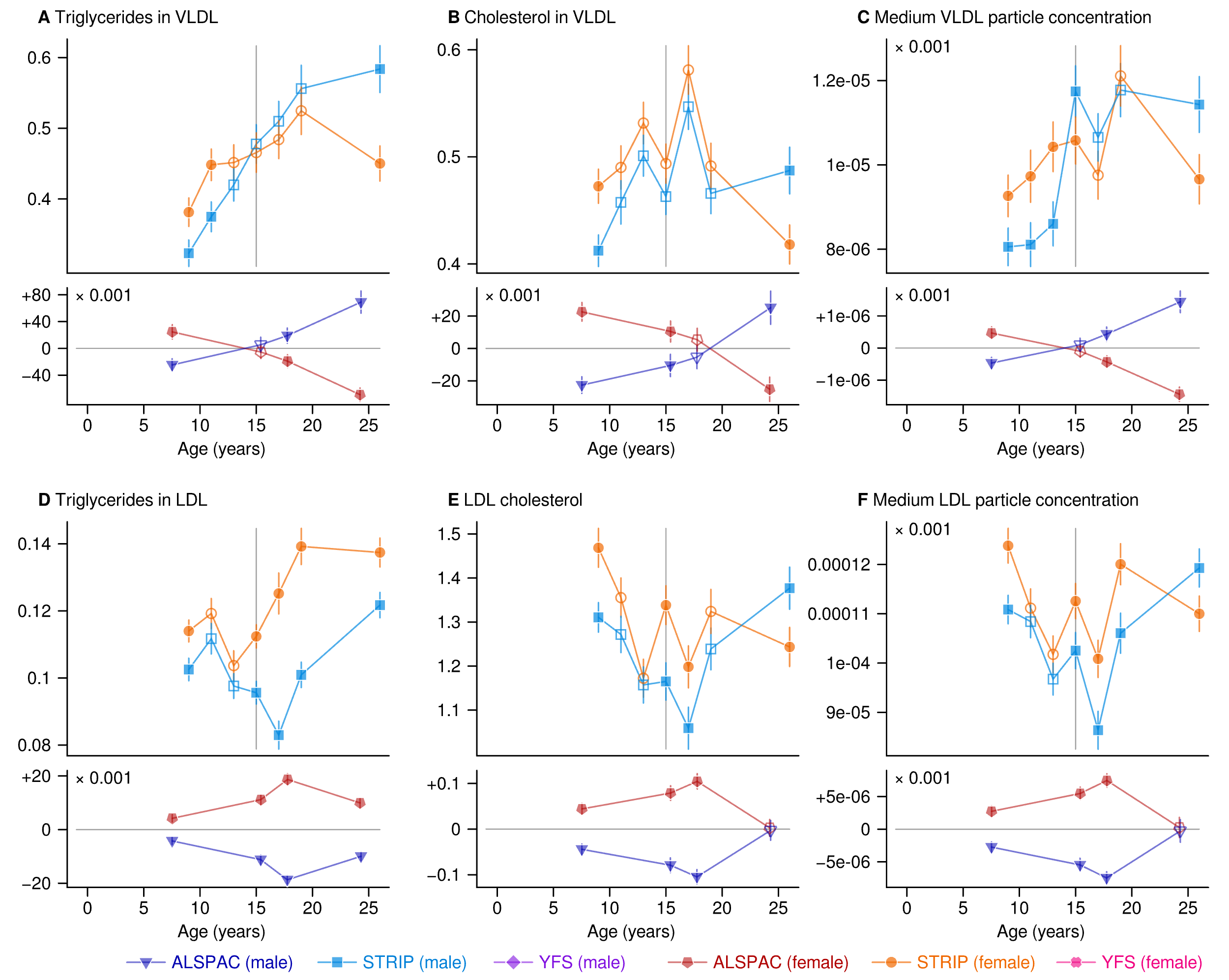
**

**Supplementary Figure S11:** Robust mean values and 95% confidence intervals for selected circulating metabolic measures in mmol/L. The results are calculated separately for each cohort and for males and females. The filled symbols indicate a statistical difference that satisfies the single-test threshold P < 0.0001. The plots consist of two subplots; the lower subplot shows the results from ALSPAC centred around the mean values of the peer groups. Abbreviations: ALSPAC (Avon Longitudinal Study of Parents and Children), LDL (low-density lipoprotein), STRIP (Special Turku Coronary Risk Factor Intervention Project), YFS (Cardiovascular Risk in Young Finns Study), VLDL (very low density lipoprotein).
